# Unlocking the secret to *Staphylococcus aureus* survival in serum

**DOI:** 10.1128/msystems.01813-25

**Published:** 2026-04-29

**Authors:** Warren E. Rose, John-Demian Sauer

**Affiliations:** 1 School of Pharmacy, University of Wisconsin-Madison 5228, Madison, Wisconsin, USA; 2 Department of Medical Microbiology and Immunology, University of Wisconsin-Madison 5228, Madison, Wisconsin, USA; Wageningen University, Wageningen, the Netherlands

**Keywords:** bacteremia, *Staphylococcus aureus*, multiomics, metabolism

## Abstract

*Staphylococcus aureus* bloodstream infections remain a major clinical challenge. A key knowledge gap is how *S. aureus* adapts to the hostile, nutrient-limited environment of human serum, where immune pressures, such as complement, antimicrobial peptides, and nutritional immunity, restrict bacterial survival. Recent investigations integrating transcriptomic, proteomic, and metabolomic data across five clinically relevant *S. aureus* lineages revealed coordinated serum-specific metabolic and stress-response adaptations (W. Mujchariyakul, C. J. Walsh, S. Giulieri, C. Cramond, et al., mSystems 11:e01183-25, 2026, https://doi.org/10.1128/msystems.01183-25). Serum triggered increased gluconeogenic and TCA-cycle activity, expanded carbohydrate, amino acid, and lipid utilization, and induction of iron-acquisition systems, nucleotide biosynthesis, and oxidative-stress defenses, while suppressing ribosome biogenesis. Functional validation confirmed key roles for carbon-metabolism genes (*gapdhB, sucA*), siderophore and iron-uptake systems (*sirA, sstD*), and the peroxide regulator *perR*. These findings highlight the metabolic resourcefulness and stress resilience that enable *S. aureus* survival and persistence despite antibiotic therapy. This work underscores the importance of multiomic approaches across pathogens and physiologic models to reveal new therapeutic targets for bloodstream infections.

## COMMENTARY

Bloodstream infections caused by *Staphylococcus aureus* are a significant cause of morbidity. The outcomes of patients with *S. aureus* bacteremia are suboptimal, with high rates of mortality and persistent infection (1). These trends continue despite new antimicrobials, rapid diagnostics, and other focused clinical approaches to improve outcomes over the last three decades. While *S. aureus* virulence factors and antibiotic resistance mechanisms are now well described, our understanding of how this opportunistic pathogen survives in human serum remains limited (2). Serum is a cell-free component of blood that is inherently hostile to bacteria, requiring *S. aureus* to adapt in ways that are different from traditional bacteria growth medium, such as cation-adjusted Mueller Hinton broth, where antibiotic susceptibility is tested (3). During bloodstream infection, *S. aureus* has several mechanisms for survival, including blocking antibody-mediated opsonization (4), degrading complement (5), and neutralizing and resisting antimicrobial peptides through surface charge modification and cell-envelope remodeling (6, 7). Further, *S. aureus* evades host-imposed nutritional immunity via trace-metal acquisition mechanisms that support its survival in blood (8). Although regulation of established virulence factors in blood has been reported, the physiological adaptations that enable *S. aureus* to survive in the bloodstream or serum remain largely undefined. An initial key step toward elucidating bacterial adaptation in serum identified commonalities among four primary sepsis pathogens, *Escherichia coli*, *Klebsiella pneumoniae* species complex, *Staphylococcus aureus,* and *Streptococcus pyogenes*. Using a multiomics approach, Mu et al. (9). previously identified broad functional patterns, including changes in fatty acid and lipid biosynthesis and metabolism and cholesterol acquisition across these pathogens in serum. Although pivotal, this study independently analyzed the multiomics response and lacked complete integration of the omics data to connect the network of the *S. aureus* biological response in serum.

To fully reveal the *S. aureus* metabolic response in serum, in recent work, Mujchariyakul et al. (10) integrate transcriptomic, proteomic, and metabolomic (GC-MS and LC-MS) analyses of five clinically relevant clonal lineages of *S. aureus* (ST1, ST5, ST22, ST239, ST8) in human serum. Multiomics analyses using the Data Integration Analysis for Biomarker discovery using Latent cOmponents (DIABLO) framework identified adaptations in human serum versus Roswell Park Memorial Institute (RPMI) medium, a cell culture medium supportive of *S. aureus* growth.

Using this multiomics approach, the five clinically relevant clonal lineages of *S. aureus* were conditioned in human serum (batch controlled, male AB plasma) and RPMI. Principal component analysis and DIABLO were applied for separation and integration of the data, respectively. Across all the clonal lineages, the isolates displayed condition-specific changes with a distinctive and coordinated *S. aureus* response. Enriched pathways (key genes mapped) in serum included cell wall biosynthesis and lipid metabolism (*glmS*), carbohydrate metabolism (*gapdhB* and *sucA*), iron transport (*sstD*, *scdA*, and *isdB*), and defense against host immunity (*perR* and *sbi*). This supports previously known metabolic reprogramming and reliance on enhanced iron uptake to maintain fitness under iron-limiting environments, such as serum (11). At the transcriptional and proteomic level, upregulation of carbohydrate transport and metabolism pathways, induction of iron-acquisition systems, activation of nucleotide biosynthesis, and simultaneous suppression of ribosome biogenesis were noted in serum. These coordinated changes highlight that enhanced metabolic flexibility, iron scavenging/homeostasis, and resource conservation are essential for *S. aureus* to survive under serum stress.

Next to validate these multiomic adaptations, Mujchariyakul et al. employed *S. aureus* transposon mutants in key genes involved in carbon metabolism, iron acquisition, and oxidative stress resistance. This was performed using the USA300 JE2 strain transposon library, which has ST8 clonal background. Utilization of targeted mutations confirmed roles for *gapdhB* and *sucA* in serum survival. Subsequent biolog metabolic analysis of these mutants revealed compound-specific roles for each gene, most notably demonstrating that methyl pyruvate could rescue *sucA*-dependent phenotypes but not *gapdhB* mutant phenotypes, highlighting a key role for gluconeogenesis in serum survival. Serum exposure also strongly induced siderophore- and catechol-based iron acquisition systems (*sirA, sstD, htsA, isdB*); however, only *sirA* and *sstD* mutants demonstrated serum survival defects, both validating the importance of these iron scavenging mechanisms for survival and highlighting the need to validate omics data with experimental loss of function data. In response to oxidative stress, *perR* mutants displayed marked sensitivity to hydrogen peroxide in serum and reduced staphyloxanthin (pigment) production.

Finally, the study provides a literature-based, comprehensive metabolic network analysis to overlay the single transcriptomics analysis and DIABLO framework for data integration. This revealed metabolic alteration in serum toward energy-efficient carbon use through upregulation of gluconeogenic and TCA-cycle enzymes and by adjusting carbohydrate metabolism around G6P/F6P, GA3P/DHAP, and pyruvate. Serum exposure also stimulated expression of virulence and immune-evasion genes, activated lipid and amino acid catabolism, and triggered oxidative-stress defense noted by enhanced *perR* regulation and staphyloxanthin production and iron–sulfur cluster repair.

This study exemplifies the difficulty in treating patients with *S. aureus* bacteremia. The extreme adaptability of *S. aureus* to adjust, survive, and thrive in nutrient-limited environments, particularly serum, presents significant challenges for antibiotic treatment. Its ability to use variable carbon sources, increase iron scavenging and iron transport, and resistance to oxidative stress revealed in this study aligns with the proclivity for *S. aureus* to persist in serum during antibiotic treatment (12, 13). While this adaptability poses challenges to treatment, the findings of this study also highlight novel areas for therapeutic intervention. The requirement for *S. aureus* to upregulate iron acquisition system suggests that inhibition of siderophores and iron transport could be a novel area for therapeutic intervention. Indeed, such strategies are currently in development with continued interest in siderophore-conjugate antibiotics and iron transport inhibitors both in clinical development (14, 15). Similarly, targeting bacterial central metabolism has long been a goal of antibiotic development (16), but the identification of key pathways that are upregulated for serum survival can focus development on pathways that are not essential for bacterial viability but are essential for virulence, thus potentially minimizing the development of antibiotic resistance to these inhibitors (17). Finally, multiomics approaches to defining mechanisms of virulence and host adaptation such as those described by Mujchariyakul et al. provide insights into potential combination therapy approaches that could simultaneously target parallel adaptations for survival in the host, thus increasing therapeutic efficacy.

While the study by Mujchariyakul et al. opens many new directions for future research in *S. aureus*, it also highlights many future directions for expanding these studies. Specifically, while their study focused exclusively on *S. aureus*, similar approaches to other bloodstream and septic infections would provide additional insights, in particular utilizing similar multiomic approaches for related (streptococci, enterococci, etc.) and unrelated pathogens (*Klebsiella* spp., *Acinetobacter* spp., etc.) would elucidate shared mechanisms of adaptations while also providing pathogen-specific approaches that could minimize negative impacts on beneficial components of the microbiome. Another key next discovery step is repeating these experiments in human plasma-like media to separate starvation effects from other serum-driven responses. Similarly, while Mujchariyakul et al. studied the adaptation to serum, the presence of innate immune cells in whole blood adds additional stress and potential mechanisms of nutrient restriction that could be important to understand in future studies. Indeed, a major mechanism of nutritional immunity in *S. aureus* infections is mediated by neutrophil calprotectin sequestration of zinc (18). Future studies adding cellular components to these multiomics approaches will continue to refine our understanding of pathogen adaptation and virulence. Finally, these studies highlight key metabolic adaptations in serum, yet how these adaptations are altered by the presence of antibiotics remains unknown. From a clinical context, the bloodstream is typically not the source of *S. aureus* infection. Rather, it is a manifestation of emboli from variable host environments, such as endocarditis, pneumonia, cellulitis, etc. Understanding how *S. aureus* adapts as it transitions from its source environment, such as endocardial vegetations, where adhesion and antibiotic inoculum effects are pronounced (19, 20), into serum is essential for guiding therapeutic strategies. Assessing antibiotic tolerance/resistance in serum with similar methods to clarify how the host environment and *S. aureus* adaptability shape treatment efficacy could further inform how serum influences antibiotic adaptation and additional potential for combination therapies that would be more effective during bloodstream infections.
